# Supplementary figures and images for: Deep learning model calibration for improving performance in class-imbalanced medical image classification tasks
Source: PLoS One. 2022 Jan 27;17(1):e0262838. doi: 10.1371/journal.pone.0262838 (PMC8794113; doi:10.1371/journal.pone.0262838)

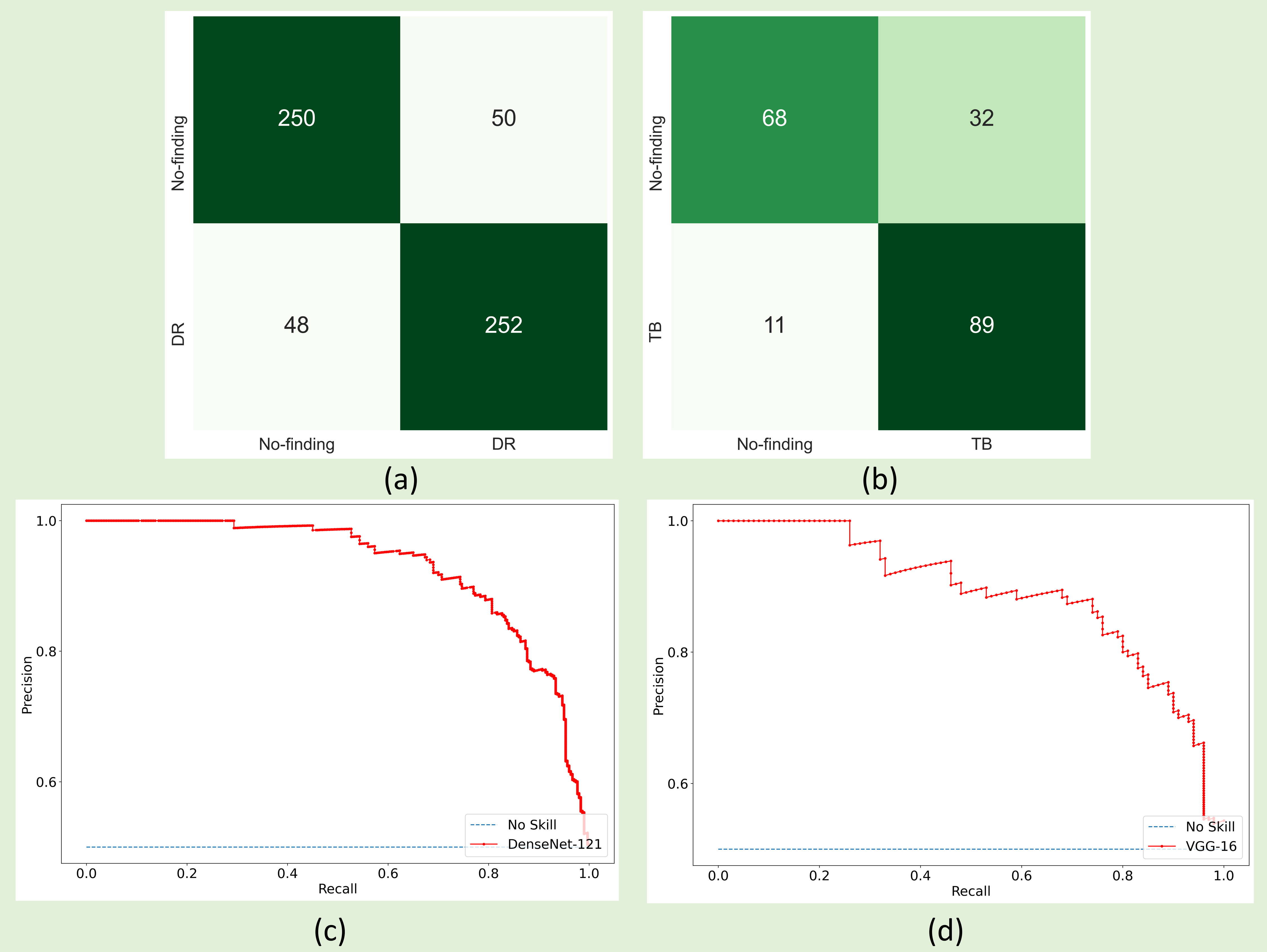

Supplement: S1 Fig — (a) and (b) confusion matrix achieved by the DenseNet-121 and VGG-16 models, respectively, using the APTOS’19 fundus and Shenzhen TB CXR data collections; (c) and (d) AUPRC curves achieved by the DenseNet-121 and VGG-16 models, respectively, using the APTOS’19 fundus and Shenzhen TB CXR data collections. (TIF) [file pone.0262838.s001.tif]

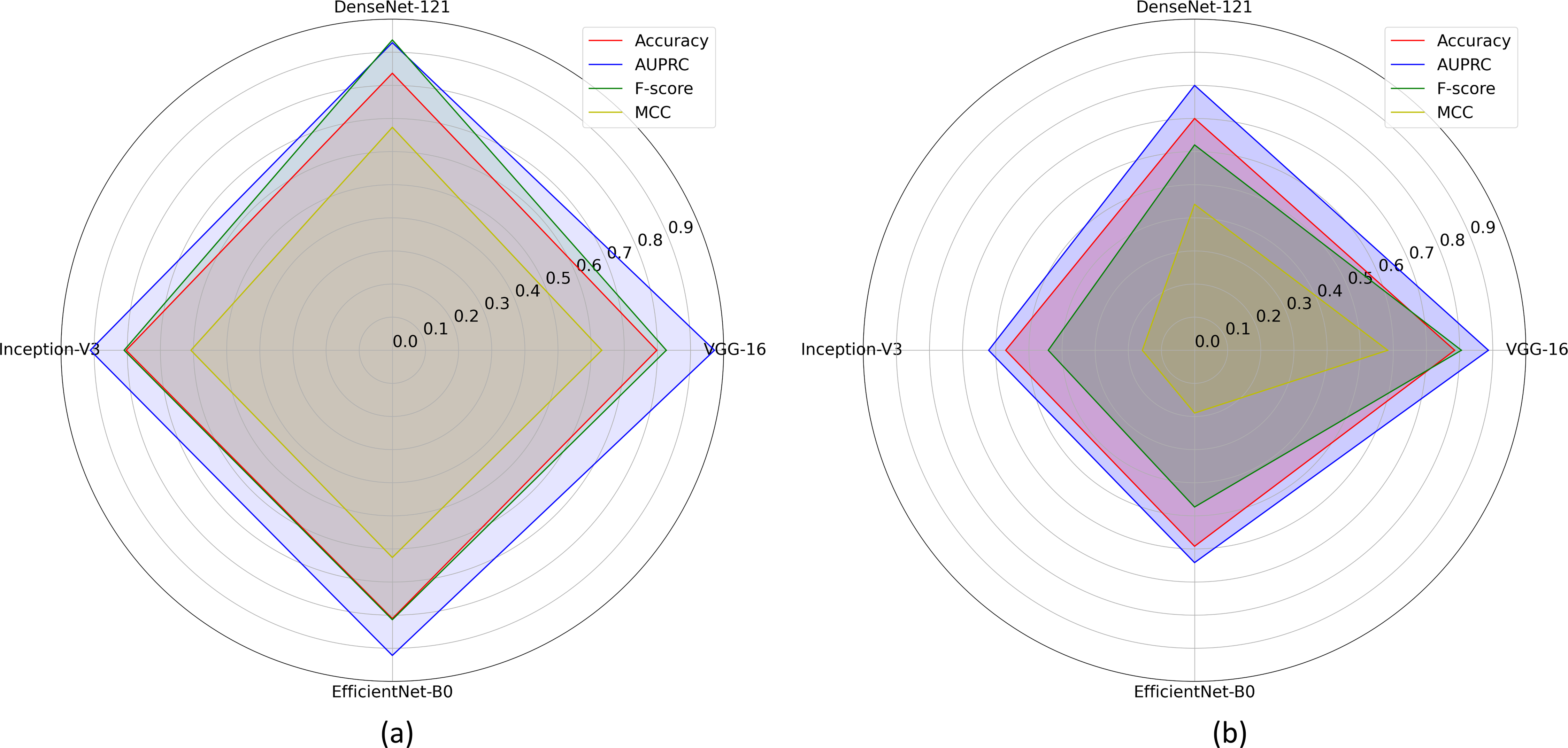

Supplement: S2 Fig — Polar coordinates plot showing the test performance achieved by the models retrained on the Set-100 dataset from (a) APTOS’19 fundus and (b) Shenzhen TB CXR datasets. (TIF) [file pone.0262838.s002.tif]

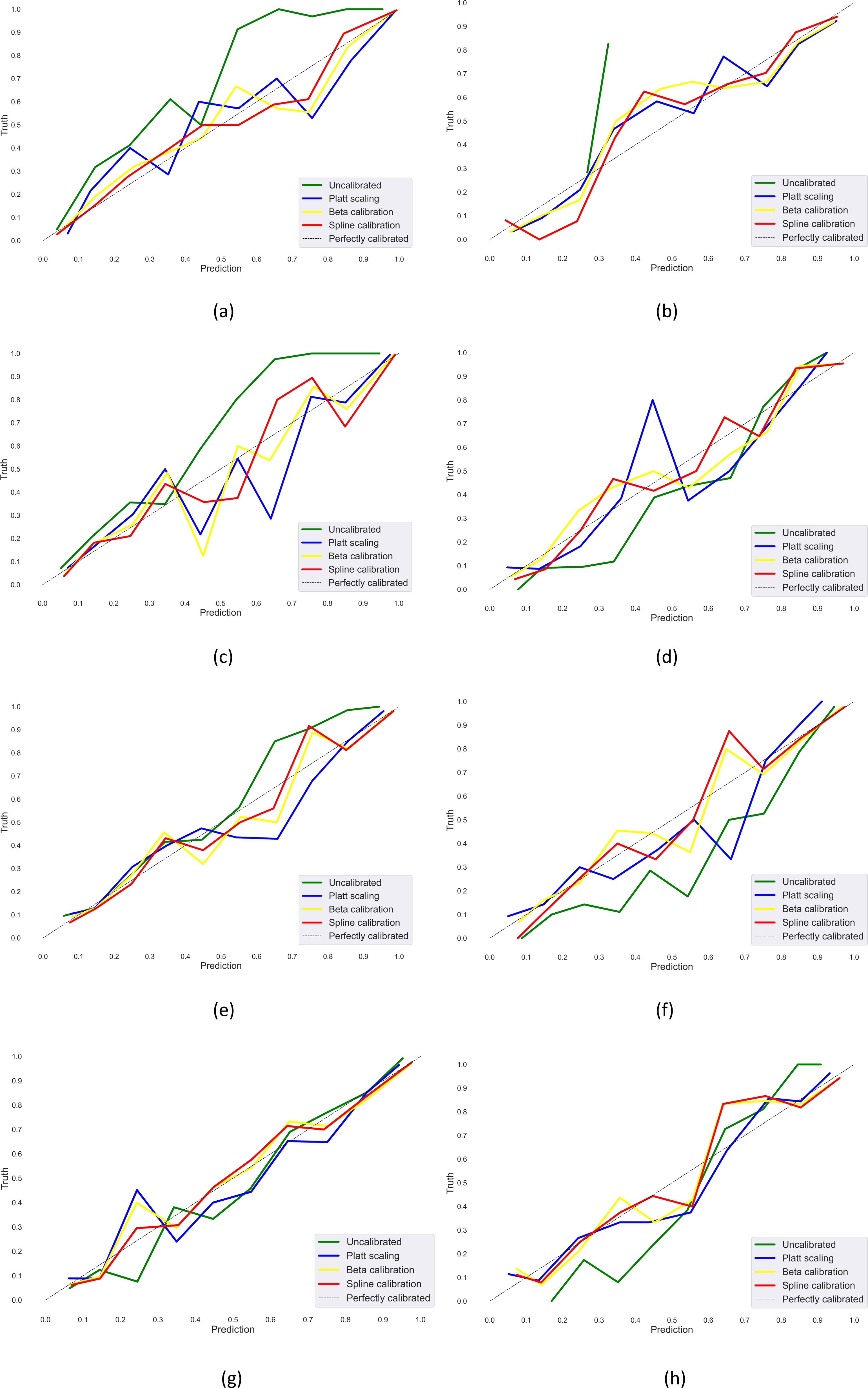

Supplement: S3 Fig — (a), (c), (e), and (g) shows the reliability diagrams obtained respectively using the. Set-40, Set-60, Set-80, and Set-100 datasets constructed from APTOS’19 fundus dataset; (b), (d), (f), and (h) show the reliability diagrams obtained respectively using the Set-40, Set-60, Set-80, and Set-100 datasets constructed from Shenzhen TB CXR dataset. (TIF) [file pone.0262838.s003.tif]

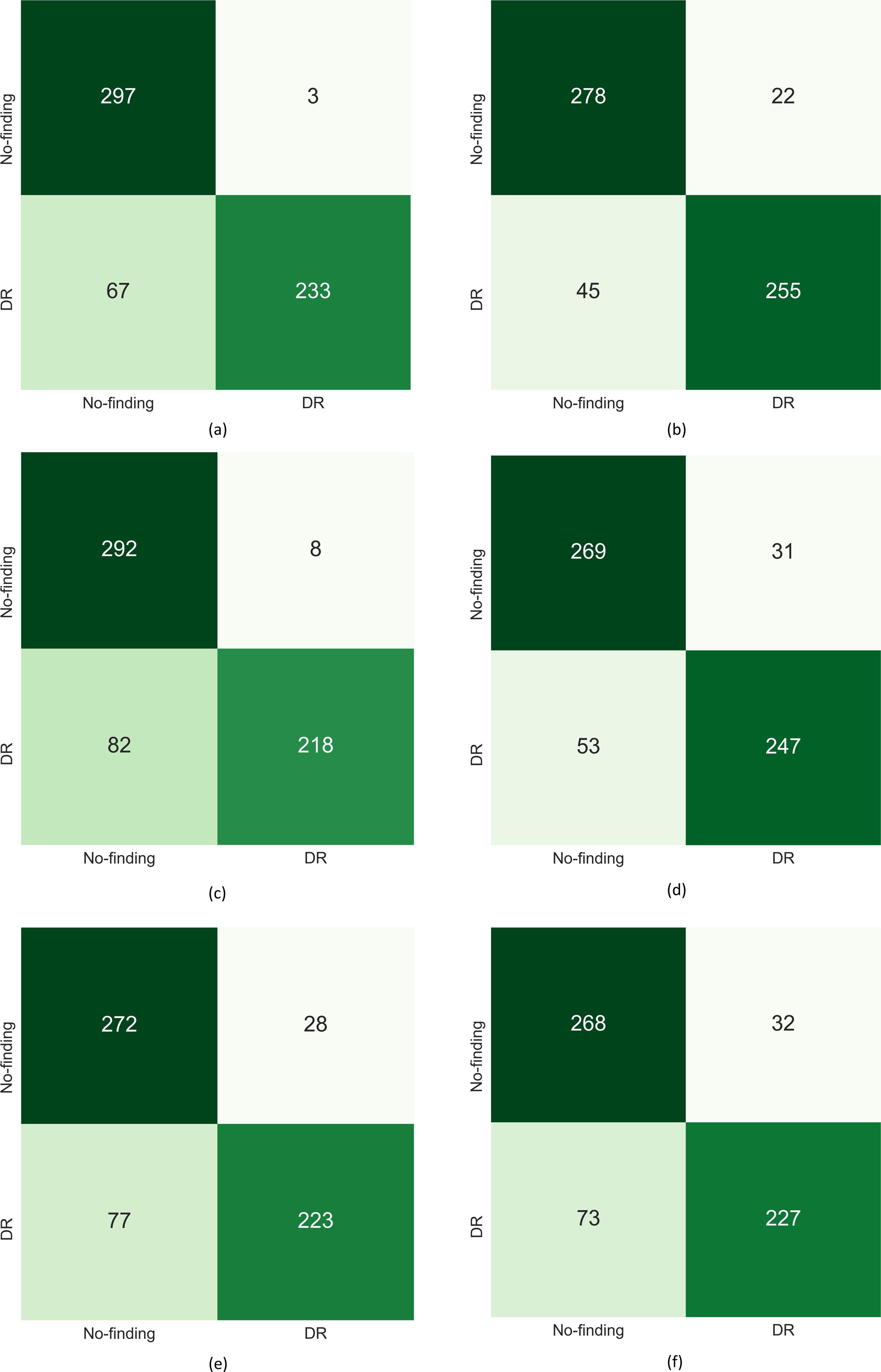

Supplement: S4 Fig — Confusion matrices obtained using the uncalibrated and calibrated probabilities (from left to right) at the baseline threshold of 0.5 for the Set-40, Set-60, and Set-80 datasets constructed from the APTOS’19 fundus dataset. (a), (c), and (e) show the confusion matrices obtained using uncalibrated probabilities; (b), (d), and (f) show the confusion matrices obtained using calibrated probabilities. (TIF) [file pone.0262838.s004.tif]

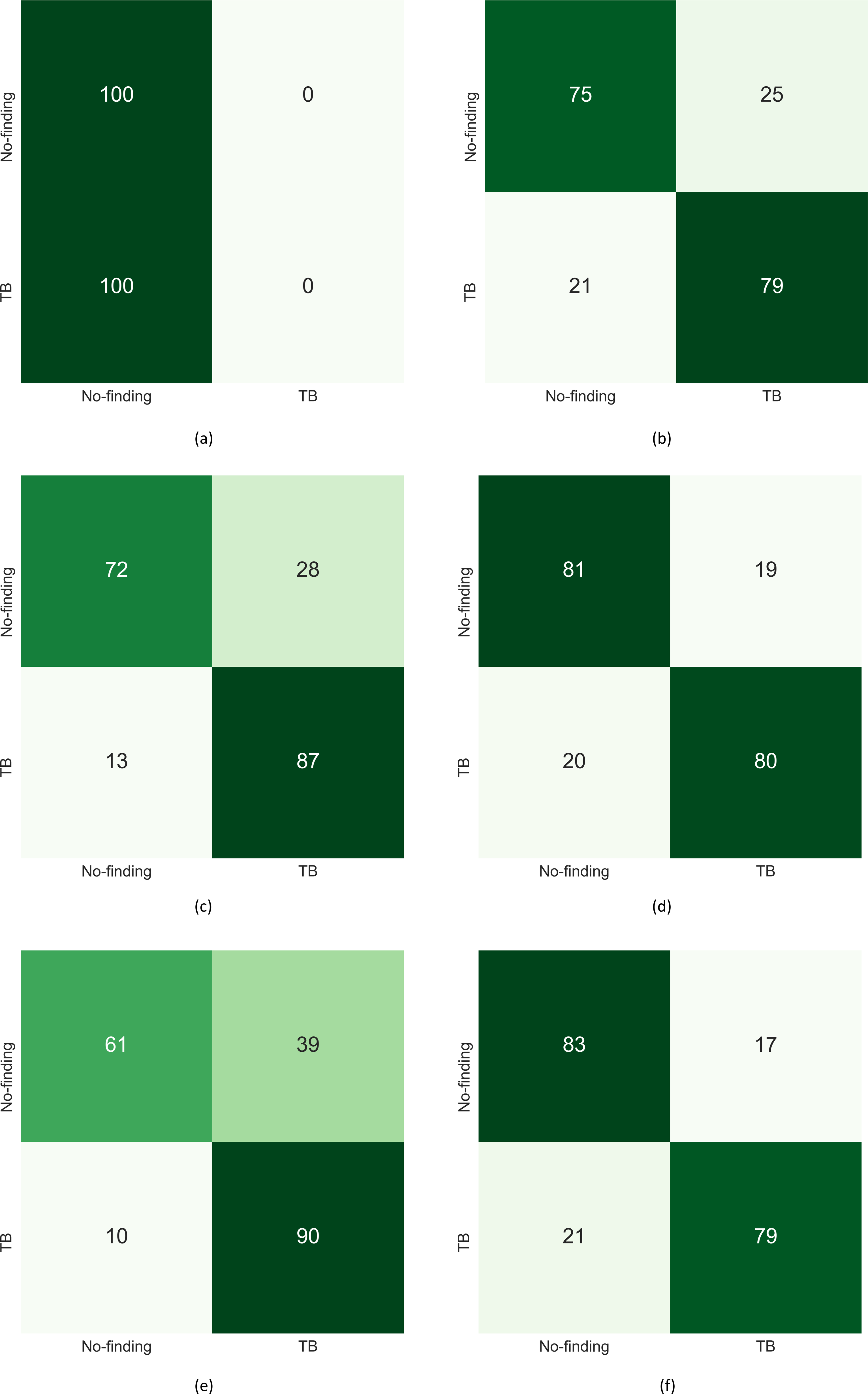

Supplement: S5 Fig — Confusion matrices obtained using the uncalibrated and calibrated probabilities (from left to right) at the baseline threshold of 0.5 for the Set-40, Set-60, and Set-80 datasets constructed from the Shenzhen TB CXR dataset. (a), (c), and (e) show the confusion matrices obtained using uncalibrated probabilities; (b), (d), and (f) show the confusion matrices obtained using calibrated probabilities. (TIF) [file pone.0262838.s005.tif]

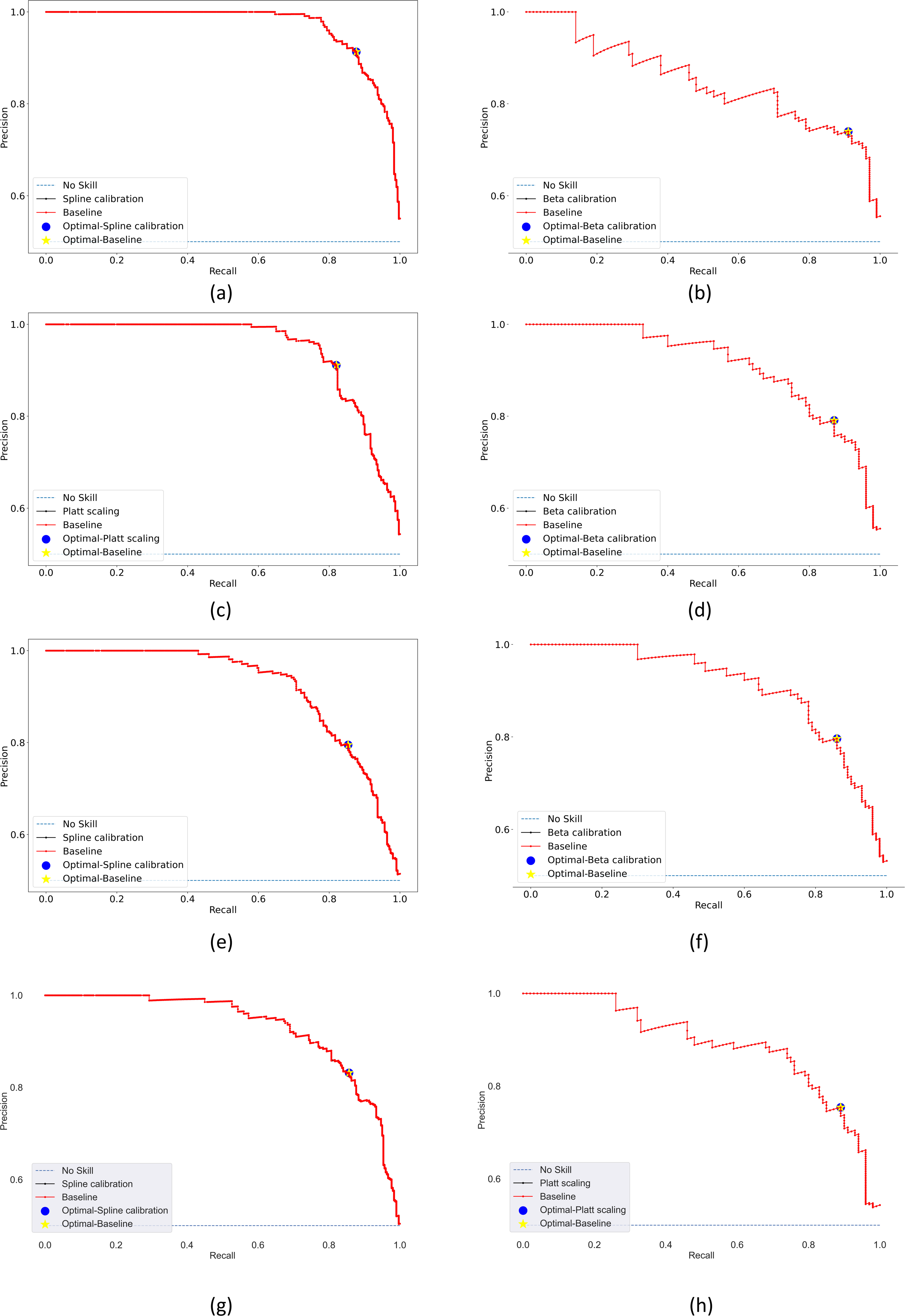

Supplement: S6 Fig — (a), (c), (e), and (g) shows the PR curves obtained respectively using the Set-40, Set-60, Set-80, and Set-100 datasets from APTOS’19 fundus dataset; (b), (d), (f), and (h) show the PR curves obtained respectively using the Set-40, Set-60, Set-80, and Set-100 datasets from Shenzhen TB CXR dataset. (TIF) [file pone.0262838.s006.tif]

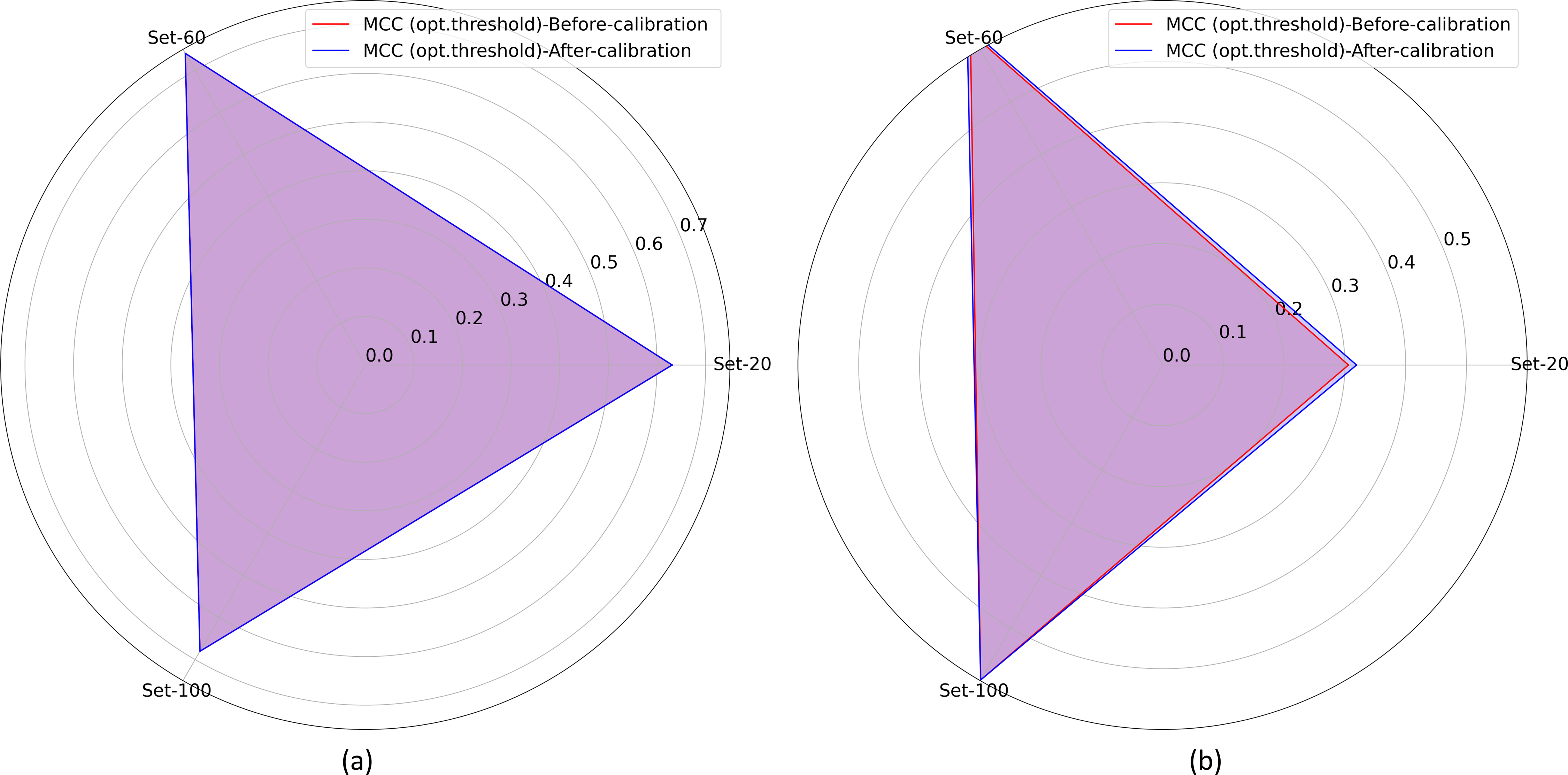

Supplement: S7 Fig — Polar coordinates plot showing the MCC metric achieved at the optimal operating thresholds, by the DenseNet-121 and VGG-16 models using calibrated and uncalibrated probabilities generated from Set-20, Set-60, and Set-100 datasets for (a) APTOS’19 fundus and (b) Shenzhen TB CXR data collections, respectively. (TIF) [file pone.0262838.s007.tif]

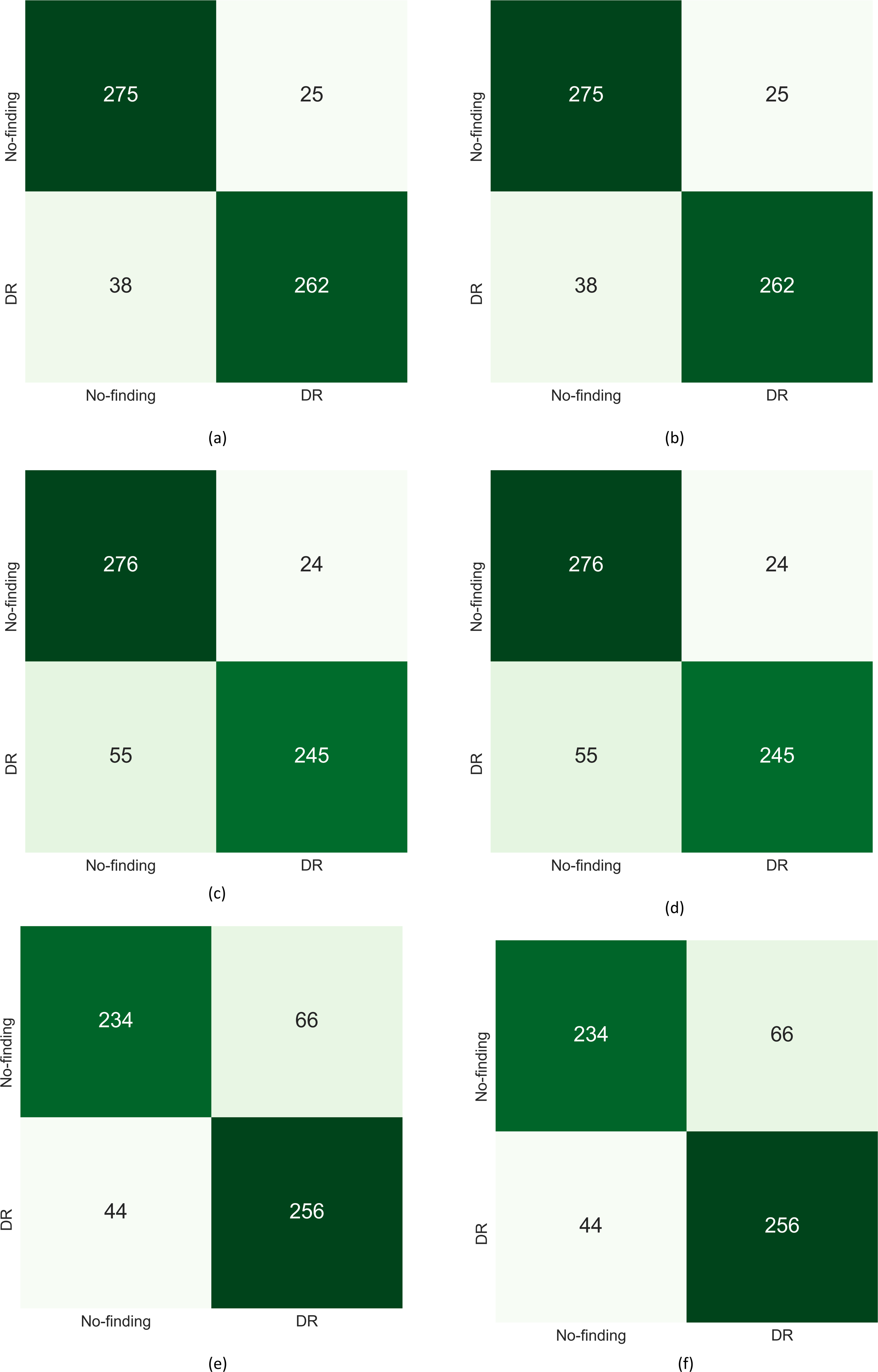

Supplement: S8 Fig — Confusion matrices obtained using the uncalibrated and calibrated probabilities (from left to right) at the optimal thresholds derived from the PR curves for the Set-40, Set-60, and Set-80 datasets constructed from the APTOS’19 fundus dataset. (a), (c), and (e) show the confusion matrices obtained using uncalibrated probabilities; (b), (d), and (f) show the confusion matrices obtained using calibrated probabilities. (TIF) [file pone.0262838.s008.tif]

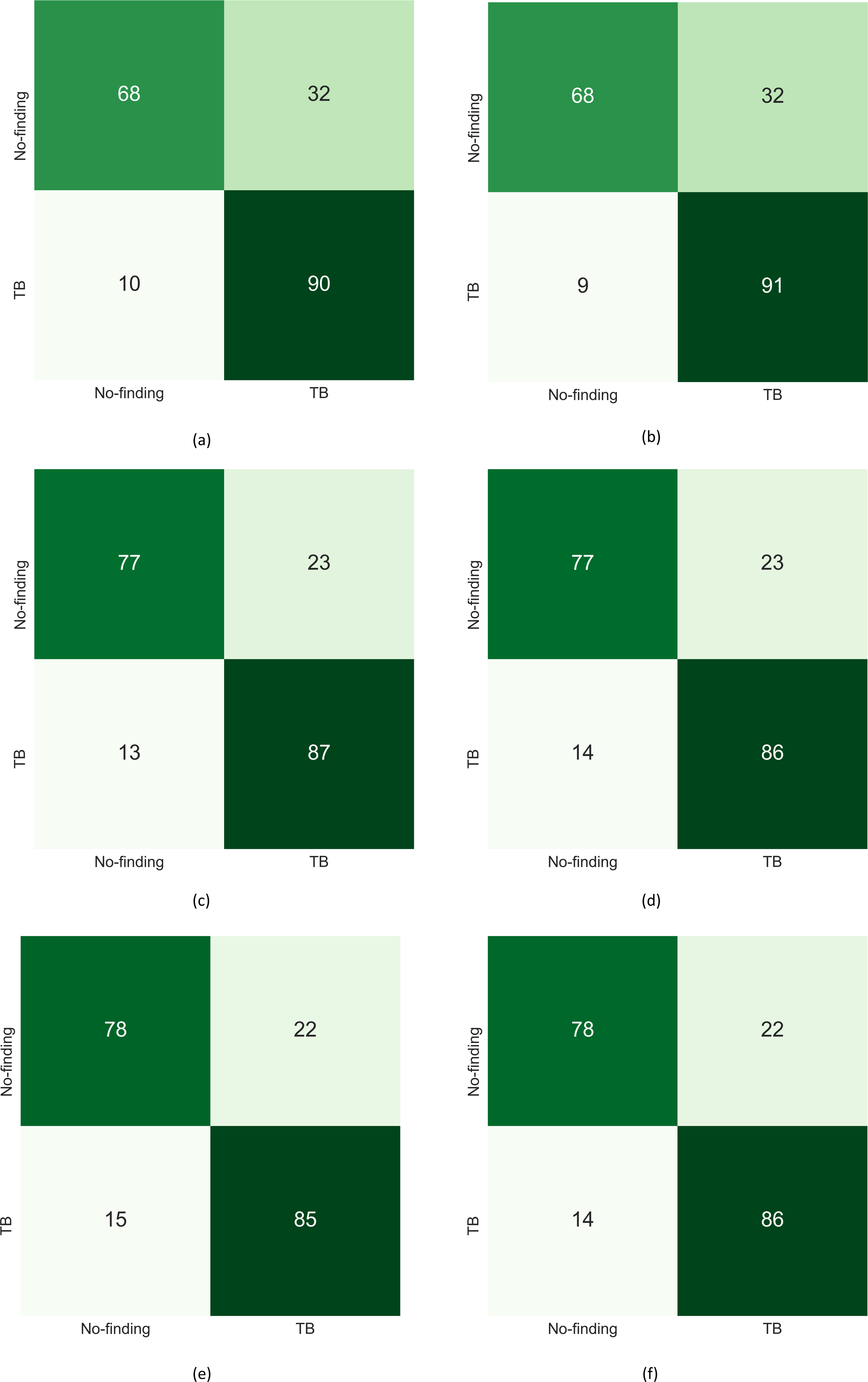

Supplement: S9 Fig. Confusion matrices obtained using the uncalibrated and calibrated probabilities (from left to right) at the optimal thresholds derived from the PR curves for the Set-40, Set-60, and Set-80 datasets constructed from the Shenzhen TB CXR dataset. (a), (c), and (e) show the confusion matrices ob — (TIF) [file pone.0262838.s009.tif]
