# Supplementary material for: Deep learning model calibration for improving performance in class-imbalanced medical image classification tasks
Source: PLoS One. 2022 Jan 27;17(1):e0262838. doi: 10.1371/journal.pone.0262838 (PMC8794113; doi:10.1371/journal.pone.0262838)
Supplement: S1 Table — The value n denotes the number of test samples. Data in parenthesis are 95% CI as the Wilson score interval provided for the ECE metric. The best performances are denoted by bold numerical values in the corresponding columns. (PDF) [file pone.0262838.s010.pdf]

Table 1. ECE metric achieved by the DenseNet-121 and VGG-16 models that are respectively retrained on the Set-40 and Set-80 datasets, individually from APTOS'19 fundus (n=600) and Shenzhen TB CXR (n = 200) image collections. The value *n* denotes the number of test samples. Data in parenthesis are 95% CI as the Wilson score interval provided for the ECE metric. The best performances are denoted by bold numerical values in the corresponding columns.

Metric	Calibration method	APTOS' 19 fundus		Shenzhen TB CXR	
		Set-40	Set-80	Set-40	Set-80
ECE	Platt	0.0464 (0.0295, 0.0633)	0.0402 (0.0244, 0.056)	0.0628 (0.0291, 0.0965)	0.0657 (0.0313, 0.1001)
	Beta	0.0258 (0.0131, 0.0385)	0.0381 (0.0227, 0.0535)	**0.0565 (0.0245, 0.0885)**	**0.0363 (0.0103, 0.0623)**
	Spline	**0.0194 (0.0083, 0.0325)**	**0.0331 (0.0187, 0.0475)**	0.0570 (0.0248, 0.0892)	0.0401 (0.0129, 0.0673)
	Baseline	0.0986 (0.0747, 0.1225)	0.0648 (0.0451, 0.0845)	0.2094 (0.1530, 0.2658)	0.1326 (0.0855, 0.1797)
