# Supplementary material for: Deep learning model calibration for improving performance in class-imbalanced medical image classification tasks
Source: PLoS One. 2022 Jan 27;17(1):e0262838. doi: 10.1371/journal.pone.0262838 (PMC8794113; doi:10.1371/journal.pone.0262838)
Supplement: S2 Table — Data in parenthesis denote the performance achieved with uncalibrated probabilities and data outside the parenthesis denotes the performance achieved with calibrated probabilities. The best performances are denoted by bold numerical values in the corresponding columns. (PDF) [file pone.0262838.s011.pdf]

Table 2. Performance metrics achieved at the baseline threshold of 0.5, by the DenseNet-121 and VGG-16 models using calibrated (using the best performing calibration method from Table 4) and uncalibrated probabilities generated for Set-40 and Set-80 datasets from the APTOS'19 fundus (n=600) and Shenzhen TB CXR (n = 200) image collections, respectively. Data in parenthesis denote the performance achieved with uncalibrated probabilities and data outside the parenthesis denotes the performance achieved with calibrated probabilities. The best performances are denoted by bold numerical values in the corresponding columns.

**Metric**	**APTOS' 19 fundus**		**Shenzhen TB CXR**	
	**Set-40**	**Set-80**	**Set-40**	**Set-80**
Accuracy	0.8883<br>(0.8833)	0.8250<br>(0.8250)	**0.7700**<br>(0.5000)	**0.8100**<br>(0.7550)
AUPRC	0.9681<br>(0.9681)	0.9208<br>(0.9208)	0.8468<br>(0.8468)	0.9069<br>(0.9069)
F-score	**0.8839**<br>(0.8694)	**0.8122**<br>(0.8094)	**0.7745**<br>(NA)	**0.8061**<br>(0.7860)
MCC	**0.7847**<br>(0.7790)	**0.6588**<br>(0.6562)	**0.5404**<br>(NA)	**0.6205**<br>(0.5329)
