# Supplementary material for: Deep learning model calibration for improving performance in class-imbalanced medical image classification tasks
Source: PLoS One. 2022 Jan 27;17(1):e0262838. doi: 10.1371/journal.pone.0262838 (PMC8794113; doi:10.1371/journal.pone.0262838)
Supplement: S3 Table — The text in parentheses shows the best-performing calibration method used to produce calibrated probabilities. (PDF) [file pone.0262838.s012.pdf]

Table 3. Optimal threshold values identified from the PR curves using uncalibrated and calibrated probabilities (using the best-performing calibration method for the respective datasets) for Set-40 and Set-80 datasets. The text in parentheses shows the best-performing calibration method used to produce calibrated probabilities.

**Data**	**APTOS' 19 fundus**		**Shenzhen TB CXR**	
	**Opt. threshold (Uncalibrated)**	**Opt. threshold (Calibrated)**	**Opt. threshold (Uncalibrated)**	**Opt. threshold (Calibrated)**
Set-40	0.2559	0.4411 (*Spline*)	0.2776	0.3458 (*Beta*)
Set-80	0.3211	0.3279 (*Spline*)	0.6338	0.3753 (*Beta*)
