# Supplementary material for: Deep learning model calibration for improving performance in class-imbalanced medical image classification tasks
Source: PLoS One. 2022 Jan 27;17(1):e0262838. doi: 10.1371/journal.pone.0262838 (PMC8794113; doi:10.1371/journal.pone.0262838)
Supplement: S4 Table — Data in parenthesis denote the performance achieved with uncalibrated probabilities and data outside the parenthesis denotes the performance achieved with calibrated probabilities. The best performances are denoted by bold numerical values. (PDF) [file pone.0262838.s013.pdf]

Table 4. Performance metrics achieved at the optimal threshold values (from Table 3), by the DenseNet-121 and VGG-16 models using calibrated (using the best performing calibration method from Table 4) and uncalibrated probabilities generated for Set-40 and Set-80 datasets from the APTOS 2019 fundus (n=600) and Shenzhen TB CXR (n = 200) datasets, respectively. Data in parenthesis denote the performance achieved with uncalibrated probabilities and data outside the parenthesis denotes the performance achieved with calibrated probabilities. The best performances are denoted by bold numerical values.

Metric	APTOS' 19 fundus		Shenzhen TB CXR	
	Set-40	Set-80	Set-40	Set-80
Accuracy	0.8950 (0.8950)	0.8167 (0.8167)	**0.7950** (0.7900)	**0.8200** (0.815)
AUPRC	0.9681 (0.9681)	0.9208 (0.9208)	0.8468 (0.8468)	0.9069 (0.9069)
F-score	0.8927 (0.8927)	0.8231 (0.8231)	**0.8161** (0.8108)	**0.8269** (0.8213)
MCC	0.7907 (0.7907)	0.6350 (0.6350)	**0.6063** (0.5946)	**0.6421** (0.6315)
